# Carbamylated Low-Density Lipoprotein (cLDL)-Mediated Induction of Autophagy and Its Role in Endothelial Cell Injury

**DOI:** 10.1371/journal.pone.0165576

**Published:** 2016-12-14

**Authors:** Chhanda Bose, Sudhir V. Shah, Oleg K. Karaduta, Gur P. Kaushal

**Affiliations:** 1 Central Arkansas Veterans Healthcare System, Little Rock, Arkansas, United States of America; 2 University of Arkansas for Medical Sciences, Department of Internal Medicine, Little Rock, Arkansas, United States of America; 3 University of Arkansas for Medical Sciences, Department of Biochemistry, Little Rock, Arkansas, United States of America; Faculty of Medicine & Health Science, UNITED ARAB EMIRATES

## Abstract

Patients with chronic kidney disease (CKD) have high risk of cardiovascular complications. Plasma levels of carbamylated proteins produced by urea-derived isocyanate or thiocyanate are elevated in CKD patients and that they are significant predictors of cardiovascular events and all-cause mortality. Carbamylated LDL (cLDL) has pro-atherogenic properties and is known to affect major biological processes relevant to atherosclerosis including endothelial cell injury. The underlying mechanisms of cLDL-induced endothelial cell injury are not well understood. Although autophagy has been implicated in atherosclerosis, cLDL-mediated induction of autophagy and its role in endothelial cell injury is unknown. Our studies demonstrate that human coronary artery endothelial cells (HCAECs) respond to cLDL by specific induction of key autophagy proteins including LC3-I, beclin-1, Atg5, formation of lipid-conjugated LC3-II protein, and formation of punctate dots of autophagosome-associated LC3-II. We demonstrated that autophagy induction is an immediate response to cLDL and occurred in a dose and time-dependent manner. Inhibition of cLDL-induced autophagy by a specific siRNA to LC3 as well as by an autophagy inhibitor provided protection from cLDL-induced cell death and DNA fragmentation. Our studies demonstrate that autophagy plays an important role in cLDL-mediated endothelial cell injury and may provide one of the underlying mechanisms for the pathogenesis of cLDL-induced atherosclerosis in CKD patients.

## Introduction

It is well established that chronic kidney disease (CKD) increases the risk for cardiovascular disease (CVD) and that end-stage kidney disease has a 10–30 times increase in cardiovascular risk than the general population [1]. Carbamylation is a nonenzymatic process of chemical modification of proteins by isocyanic acid generated upon dissociation of urea and by the myeloperoxidase-catalyzed oxidation of thiocyanate [2,3,4]. In this process isocyanic acid reacts irreversibly with free amino groups and ε-NH2 of lysine residues in proteins [3, 5]. In response to a decline in renal function in uremic patients, accumulation of urea concentrations results in increased levels of isocyanic acid in the blood [6] that promote carbamylation of proteins. High levels of carbamylated LDL (cLDL) have been identified in the plasma of uremic patients compared to the plasma of humans with normal kidney function [7,8,9]. Two separate clinical studies involving 1000 subjects revealed that protein-bound homocitrulline (carbamyl-lysine) independently predicted the risk for acute coronary disease or stroke, frequency of death, and frequency of major cardiovascular events [4]. In patients on hemodialysis, the highest tertile of protein carbamylation was associated with a significant higher mortality, and Kaplan-Meier analyses revealed a significant association between elevated protein carbamylation and death over a 5-year follow-up period [9]. In the Accelerated Mortality on Renal Replacement (ArMORR) study, patients who died within 12 months had significantly higher protein carbamylation compared to patients who survived the 12-month period [10]. Similarly, a significant risk of death among 4D subjects was reported with elevated carbamylated albumin [10]. A recent study from 1161 diabetic patients on hemodialysis revealed association of carbamylated albumin with congestion heart failure and sudden cardiac death [11]. In patients with CKD, LDL carbamyl-lysine levels were significant predictors of cardiovascular events and all-cause mortality [12].

Our studies have demonstrated that cLDL affects major biological processes relevant to atherosclerosis including endothelial cell injury and vascular smooth muscle cell proliferation [7, 13,14]. Although endothelial cell injury is initially involved in the pathogenesis of atherosclerosis [15,16] the underlying mechanisms by which cLDL induces endothelial cell injury are not known. Autophagy is a conserved multistep process of degradation of proteins, organelles, and other macromolecules by the lysosome [17,18]. The degraded cellular contents are recycled to synthesize new macromolecules and organelles. A low level of basal autophagy occurs under normal physiological conditions to maintain cellular homeostasis [17,18,19]. Under stress conditions of cell starvation, hypoxia, nutrient- and growth-factor deprivation, oxidant injury, and other damaging insults, autophagy induction generally promotes an adaptive or survival role [20,21,22,23]. Under certain conditions, excessive autophagy or dysregulated autophagy may contribute to cell death [24,25,26]. Although autophagy has been implicated in atherosclerosis, cLDL-mediated induction of the autophagy pathway and its role in endothelial cell injury has not been previously investigated. It is not known whether cLDL-mediated endothelial cell injury involve autophagy. In the present study we examined the induction and role of autophagy in cLDL-induced endothelial cell injury by utilizing complementary pharmacological and genetic approaches.

## Materials and Methods

### Cell culture

Human coronary artery endothelial cells (HCAECs) were purchased from Lonza (Walkersville, MD) and used at passages between 4 and 6. Cells were cultured and maintained in endothelial growth medium microvasculature (EGM-2-MV; Lonza), supplemented with growth factors and 5% fetal bovine serum (FBS),100 U/mL penicillin, 100 μg/mL streptomycin, and maintained at 37°C in a humidified incubator (5% CO_2_). For experiments cells were treated with 25 to 400 μg/mL LDL isoforms in serum-free EGM-MV medium for 1 to 24 hours. Control cells were treated with PBS for the same period of time.

### Preparation of cLDL

Human native LDL (nLDL) and all other chemicals were purchased from Sigma-Aldrich (St. Louis, MO) unless stated otherwise. Carbamylated LDL was prepared by the method of Weisgraber et al. as we previously described [7]. Briefly, sterile potassium cyanate (KOCN; Aldrich, Milwaukee, WI) was added to the lipoprotein solution at 20 mg/mg LDL protein. The mixture was incubated at 35°C for 4 hours. KOCN was removed by excessive dialysis under sterile conditions at 4°C against 0.15 mol/L NaCl, 0.01% ethylenediaminetetraacetic acid (EDTA), pH 7.0, for 36 hours. About 5 mL of the LDL preparation was dialyzed against 5 L buffer, which was changed every 12 hours. After modification, LDLs were dialyzed separately against the same buffer. The dialysis buffer after the second or third dialyses had neither a cytotoxic nor proliferative effect on cells in control experiments. A colorimetric method using diacetyl monoxime was used to measure the degree of carbamylation in LDL preparations [27]. The electrophoretic mobility of nLDL and cLDL was determined in 0.5% agarose gel, 0.2% bovine serum albumin (w/v) as described by Noble [28]. A standard curve was generated using homocitrulline (e-amino-carbamyllysine, 0 to 30 nmol) (Advanced Asymmetrics, Inc. Millstadt, IL, USA). The results were expressed in nmol homocitrulline/mg LDL protein. All LDL isoform preparations were adjusted to 1 g protein/L with PBS containing 200 μmol/L EDTA, kept at 4°C away from light, and used within 2 weeks after preparation. If sediment appeared during storage, it was removed by low-speed centrifugation, and only soluble fractions of the LDL modifications were used for experiments.

### Western blot analysis

Total cell extracts were analyzed by SDS-PAGE utilizing 4–20% Tris-glycine separating gel (Invitrogen Life Technologies; Carlsbad, CA). Monoclonal rabbit anti-LC3 antibody, diluted to 1:1,000 and horseradish peroxidase-coupled anti-IgG, dilution 1:2,000, HRP conjugated anti-rabbit monoclonal β-actin or GAPDH, dilutions 1:2,000 (All from Cell Signaling Technology), were used for WB. For visualization of the bands Enhanced Chemiluminescence (SuperSignal West Pico Chemiluminescent Substrate; Thermo Scientific, Rockford, IL) was used. Bands were quantified using an Alpha Innotech ChemiImager 5500 (Alpha Innotech Corporation, Santa Clara, CA).

### Immunofluorescence study

Cells were seeded in 4-well BD chamber slides (Fisher Scientific) at a density of 25 x 10^3^ cells/chamber in complete growth medium and treated as above. Immunofluorescence studies were performed to check the expression of LC3. After treatment, cells were washed with 1xPBS, and fixed with cold methanol (for LC3) for 15 min at -20°C and subsequently with 4% neutral buffer formaldehyde for 15 min at room temperature. Slides were washed with 1x PBS three times for 5 min each. Nonspecific sites were blocked by incubating the cells in 0.1% triton x-100 and 5% goat serum for 30 min at room temperature and washed 3 times with 1 x PBS for 5 min each. Cells were incubated with monoclonal rabbit anti-LC3 antibody (Cell Signaling Technology, Inc., Danvers, MA), in 1% BSA and 0.1% triton x-100 in 1x PBS, at 37°C for 1h. Cells were washed 3 times with 1x PBS for 5 min each. Alexa Fluor 488 anti-rabbit IgG (Molecular Probes; Cell Signaling Technology) secondary antibody, diluted in 5% goat serum in 1x PBS was added to the cells, and incubated at 37°C for 1 h. For negative controls, cells were incubated with IgG only without specific primary antibodies. Cells were washed 5 times with 1x PBS for 5 min each and mounted with Vectashield mounting media (Vector Laboratories, Burlingame, CA) containing DAPI for counterstaining the nuclei. Slides were observed and photomicrographed under a fluorescence microscope (Nikon, ECLIPSE TE 300).

### Small-interfering RNA (siRNA) transfection

Human LC3-specific siRNA against LC3β were purchased from Santa Cruz Biotechnologies Inc. (Santa Cruz Biotechnology, Inc., Dallas, TX). For transfection, 2 x 10^5^ cells per well in a six-well tissue culture plate were seeded with 2 mL antibiotic-free growth medium supplemented with 5% FBS. Cells were incubated at 37°C in a CO_2_ incubator until the cells were 60–80% confluent. Transient transfection was performed by using the manufacturer’s protocol as we have performed previously [29]. Briefly, cells were transfected with 3 siRNA duplexes against LC3β, using 6 μL of siRNA transfection reagent (sc-29528) to a final concentration of 20–80 pmols siRNA per duplex, in 100 μL of transfection medium (sc-36868) for 8 h, followed by addition of complete media for 48 h. Control siRNA (sc-37007) containing scrambled sequence were used for negative controls. Efficiency of transfection was checked by western blotting.

### Cell toxicity assay by LDH release after cLDL treatment

To determine cellular toxicity after cLDL treatment, 6x10^3^cells were plated in 96-well tissue culture plates in EGM-2-MV medium supplemented with 5% FBS per well. After overnight for an adequate attachment, cells were washed with 1XHBSS, transferred to a serum-free EGB-2-MV medium, and treated with different doses of cLDL and nLDL (25, 50, 100, 200 and 400 μM) for 8 h. For inhibitor studies, cells were pretreated with 5mM of 3 methyladinine (3MA) (Sigma) for 1h before adding nLDL or cLDL and for transfection effect, cells were transfected with LC3 for 48h as mentioned above and used. Cell culture medium was analyzed for the percentage of lactate dehydrogenase (LDH) released using a commercially available colorimetric kit, the CytoTox 96 Non-Radioactive Cytotoxicity Assay (Promega, Madison, WI) according to the manufacturer’s instructions. Briefly, the supernatant medium (50 μL) was transferred to another 96-well plate and 50 μL of CytoTox 96 reagent was added to each well. The plate was incubated at room temperature for 30 min in the dark. 50 μl of stop solution was added to the wells and absorption was assayed at 490 nm as described above. Medium, volume correction, and spontaneous LDH release controls were applied. The cytotoxicity was expressed as the ratio of absorption of released LDH to that of the total LDH.

### TUNEL assay

A terminal deoxynucleotidyl transferase dUTP-mediated nick-end labeling (TUNEL) assay was utilized to assess and validate apoptotic cell death. TUNEL staining for FACS analysis was performed using an *in situ* cell death detection kit (Roche Diagnostics Corp., Indianapolis, IN), following the manufacturer’s protocol. Briefly, 200X10^3^ cells in 100 mm tissue culture dishes were grown in growth medium up to 70–80% confluency. Transfection and treatments with cLDL and inhibitors were performed as above. Cells were washed with PBS and fixed in freshly prepared 4% paraformaldehyde in PBS (pH 7.4), followed by incubation in permeabilization solution (0.1% Triton X-100, 0.1% sodium citrate) for 2 min on ice. Cells were rinsed twice with PBS and incubated in a TUNEL reaction mixture for 60 min at 37°C in the dark in a humidified chamber. TdT was replaced by labeling solutions as the negative controls. For positive controls, fixed and permeabilized cells were incubated with DNase I (3000 U/mL) cells for 10 min at RT in 50 mM tris- HCl containing 1% BSA. Cells were rinsed three times with PBS. For FACS analysis after incubation with TUNEL reaction mixture, cells were washed three times with ice- PBS, suspended in 250 μL of PBS and were analyzed with the FACS Calibur system (Becton, Dickinson and Co., Franklin Lakes, NJ). The fluorescence level for discrimination between apoptotic and nonapoptotic cells was set using the control without TdT. Cells above this fluorescence value in the TdT-positive sample were considered apoptotic. The percentages of cells undergoing apoptosis were assessed. Analysis was performed using the Flow Jo software (Becton, Dickinson and Co.). Data are shown as a logarithmic histogram and expressed as fluorescence intensity of number of counts of the TUNEL-positive cells obtained from the statistical analysis of the fluorescence height and mean value of the x-axis displayed by the software. Data were obtained from flow cytometry analyses from three independent experiments.

### Statistical analysis

All analyses were performed using Prism 6.0 (GraphPad Software Inc., San Diego, CA). Data from at least three independent experiments were used for statistical analysis. Results are shown as mean ± SD. Analysis of variance (ANOVA) with Tukey’s post-hoc analysis was used when >2 groups were compared. For comparison of means between two groups, two-tailed unpaired *t* test were performed. A *P* value <0.05 was considered significant.

## Results

**Fig 1:**
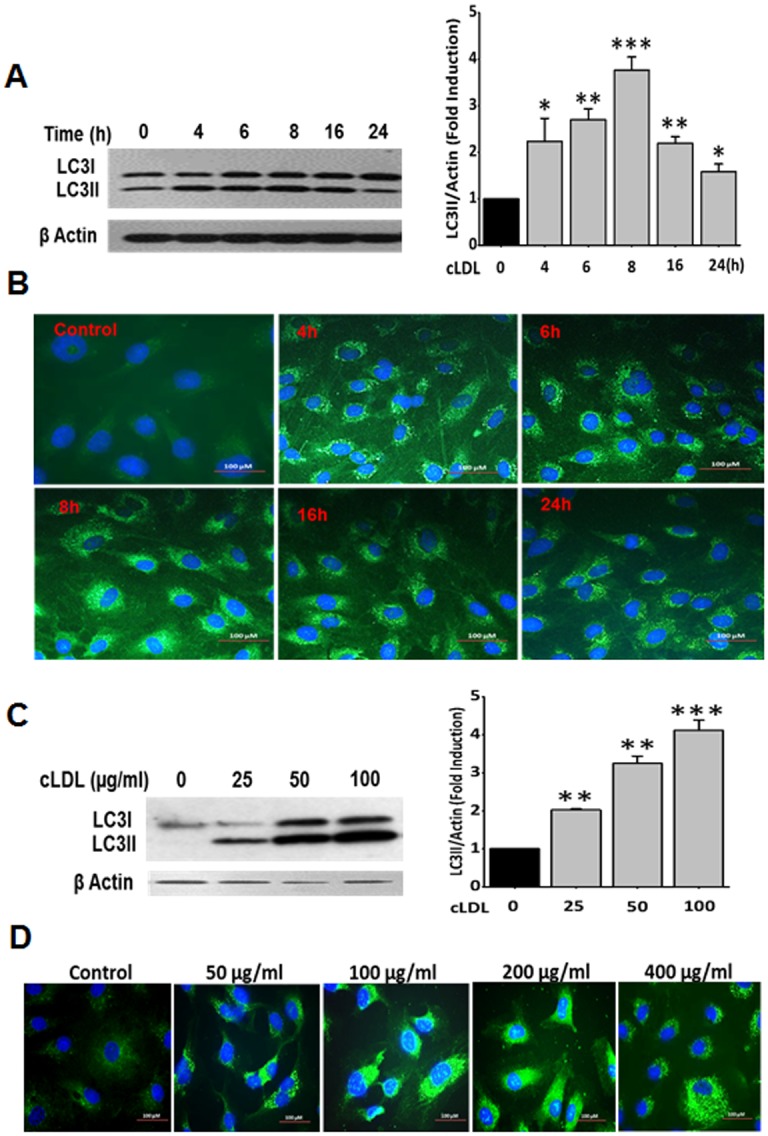
cLDL induces LC3-II formation in HCAE cells. **(A)** Time-course of cLDL-induced LC3-II formation shown by western blot analysis and **(B)** immunofluorescence staining for LC3. HCAE cells were treated with 100 μg/ml of cLDL and collected at different time points and processed for western blots and immunofluorescence staining as described in Method section. **(C)** Dose response of CLDL for LC3-II formation shown by western blot analysis, and **(D)** immunofluorescence staining withLC3 antibody in HCAE cells treated for 8h. Representative blots and images from three separate experiments are presented here. Histogram shows the quantitative analysis of western blots from three separate experiments (A right panel and C right panel). β-actin was used as a loading control for total cellular proteins. Significance of the data was determined by ANOVA, followed by paired-group comparisons. Values are presented as mean ± SD of three separate experiments (*p <0.05), **p <0.01, ***p <0.001 as compared to untreated control cells).

**Fig 2:**
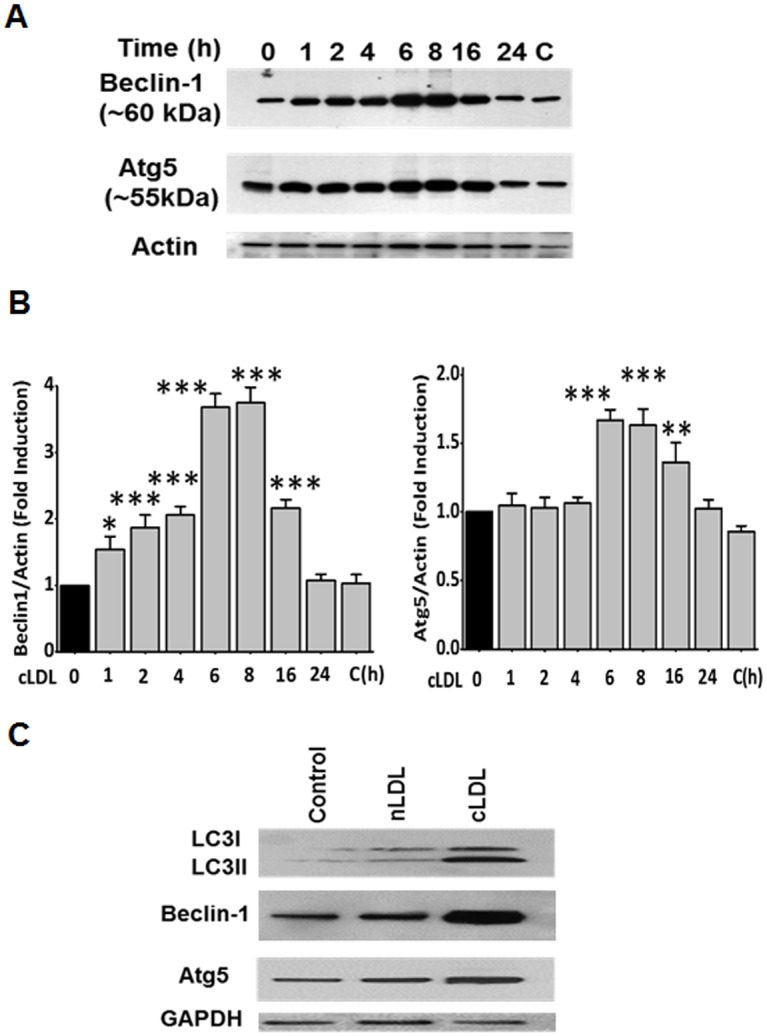
Upregulation of autophagy proteins in response to cLDL treatment. **(A left panel)** HCAE cells were treated with 100 μg/ml cLDL for various times as shown. A representative western blot for expression of autophagy proteins LC3-II, beclin-1, and Atg5 is shown. β-actin was used as a loading control. **(B)** Quantitative analysis for western blots of Fig 2A are shown in the Fig 2B (left and right panels) for beclin-1 and Atg5, respectively. Values are means ±SD, obtained from 3 separate experiments, Significance of the data was determined by ANOVA, followed by paired-group comparisons (**p <0.05), **p <0.01, ***p <0.01 as compared to untreated controls). **(C)** cLDL but not nLDL induces autophagy proteins. HCAE cells were treated with 100 μg/ml cLDL or 100 μg/ml nLDL for 8 hours and expression of autophagy proteins was determined by western blots. A representative blot from three separate experiments for the LC3-II, beclin-1, and Atg5 is shown. As shown there was no induction of autophagy proteins with nLDL in comparisons to cLDL treated cells. GAPDH was used as a loading control.

The induction of autophagy in HCAECs was determined by western blots as well as immunofluorescence staining for the formation of LC3-II from the cytosolic LC3-I. Western blot analysis indicated that cLDL treatment significantly increased LC3-II formation in a time- and dose-dependent manner (Fig 1A and 1C). LC3-II formation in response to cLDL was maximum at 8h when 100 μg/ml cLDL was used. Immunofluorescence staining with LC3 antibody showed that cLDL treatment increased LC3-II punctate dots in a dose- and time-dependent manner (Fig 1B and 1D). Maximum punctate staining for LC3-II was obtained with 100 μg/ml cLDL incubated for 8h time period similar to the formation LC3-II determined by western blot. Other key autophagy proteins, beclin-1 and Atg5 were also evaluated in endothelial cells upon treatment with cLDL. Beclin-1 and Atg5 levels were significantly increased in a time-dependent manner up to 16h (Fig 2A and 2B **left and right panel**). While cLDL induced LC3-II, beclin-1, and Atg5 levels, unmodified normal LDL (nLDL) was unable to increase the levels of autophagy proteins (Fig 2C). Taken together, these studies provide evidence that cLDL induces autophagy in endothelial cells.

**Fig 3:**
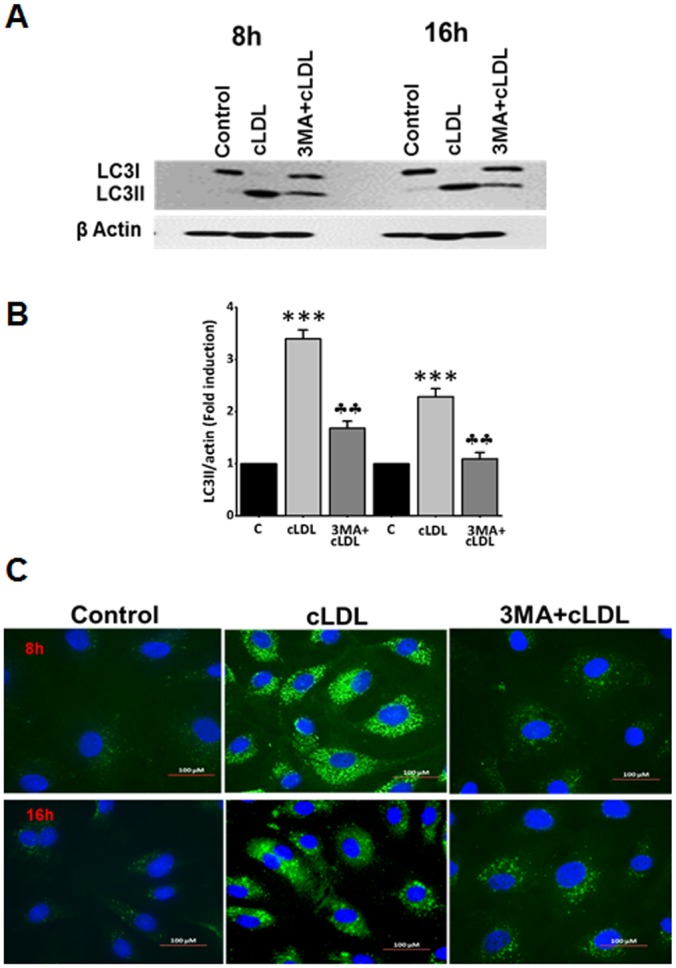
Inhibition of cLDL-induced autophagy by 3-MA. **(A)** Western blot analysis of inhibition of cLDL-induced autophagy by 3-MA. HCAE cells were treated with 100μg/ml cLDL for 8h & 16h as shown. For autophagy inhibition, cells were pretreated with 5mM 3MA for 1h before the addition of cLDL. cLDL-induced LC3-II formation was significantly inhibited by 3MA as shown by western blot analysis. β-actin was used as a loading control for total cellular proteins. A representative blot from three separate experiment is shown. **(B)** Quantitation of the bands of western blots shown in Fig 3A. Significance of the data was determined by ANOVA, followed by paired-group comparisons. Values are presented as mean ± SD of three separate experiments (***p <0.001 compared to control, ††*p* <0.001 compared to cLDL treated cells). **(C)** Immunofluorescence staining for inhibition of cLDL-induced autophagy by 3-MA. Cells were pretreated with 5mM 3MA before the addition of 100 μg/ml of cLDL for 8 and 16h as shown. Immunofluorescence staining was performed as described in Methods. 3-MA considerably reduced cLDL-induced punctate structures. The images for LC3 staining are from representatives of 3 independent experiments.

**Fig 4:**
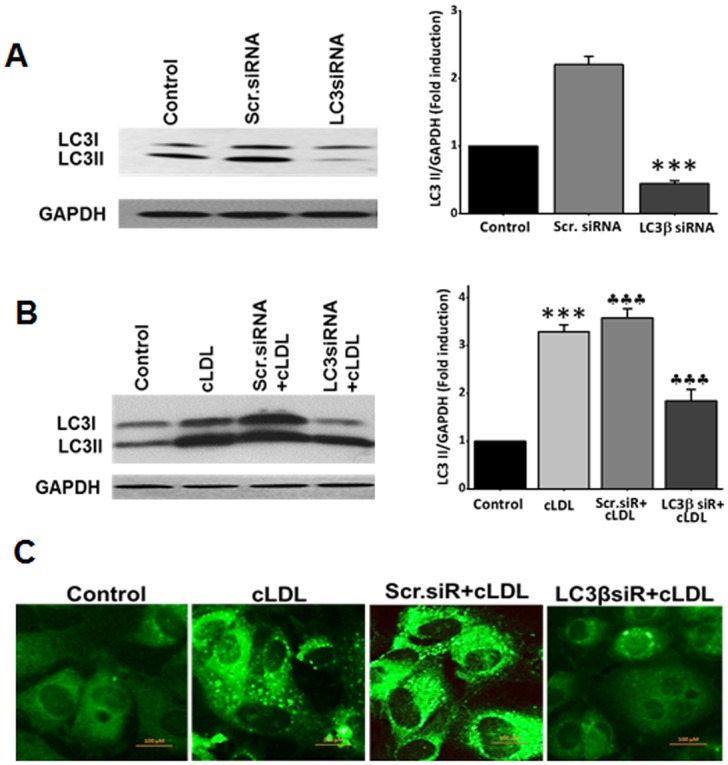
Effect of LC3 siRNA transfection on cLDL-induced autophagy. **(A)** A representative western blot in the left panel shows the efficacy of transfection with siRNA. HCAE cells transfected with LC3βsiRNA or scr.siRNA for 48h and expression of LC3 was determined by western blots. GAPDH was used as a loading control for total cellular proteins. Histograms in the right panel shows the quantitative data (mean ± SD) for western blot analysis from three separate experiments (***p <0.001 compared to control). **(B)** LC3β/scr.siRNAs transfected or untransfected cells were treated with 100μg/ml of cLDL for 8h. A representative blot for western blots is shown in the left panel. Histograms in the right panel shows the quantitative data (mean ± SD) for western blot analysis from three separate experiments. GAPDH was used as a loading control for total cellular proteins (***p <0.001 compared to control, ††*p* <0.001 compared to cLDL treated cells). **(C)** Effect of LC3βsiRNA on cLDL-induced autophagy by immunostaining. Representative images of LC3 immunostaining from three separate experiments for LC3β/scr. siRNAs transfected, cLDL treated and untreated cells are shown in the figure.

The conversion of LC3-I to LC3-II upon treatment of HCAECs with cLDL was significantly inhibited by 3-methyladenine (3-MA) (Fig 3A and 3B). Also, formation of cLDL-induced LC3-II vesicles (depicted as punctate dots) detected by immunofluorescence staining were markedly inhibited by 3-MA (Fig 3C). The autophagy inhibitor was equally effective in preventing cLDL-induced LC3-II formation at 8 h and 16 h time points tested in these experiments (Fig 3A, 3B and 3C). In addition, we used a genetic approach to examine the inhibition of cLDL-induced autophagy by utilizing siRNA to LC3. We first determined that the effect of LC3 siRNA is specific for LC3 inhibition. As shown in Fig 4A **left and right panel**, transfection of HCAECs with LC3 siRNA significantly inhibited expression of LC3. This inhibition is specific to LC3 siRNA since scrambled siRNA did not decrease the expression of LC3. LC3 siRNA significantly decreased cLDL-induced LC3-II formation compared to the scrambled LC3 siRNA (Fig 4B **left and right panel**). Also, the formation of LC3-II punctate staining was markedly reduced in HCAECs upon transfection with LC3 siRNA (Fig 4C). These studies provide evidence that 3-MA and LC3 siRNA inhibit cLDL-induced autophagy.

**Fig 5:**
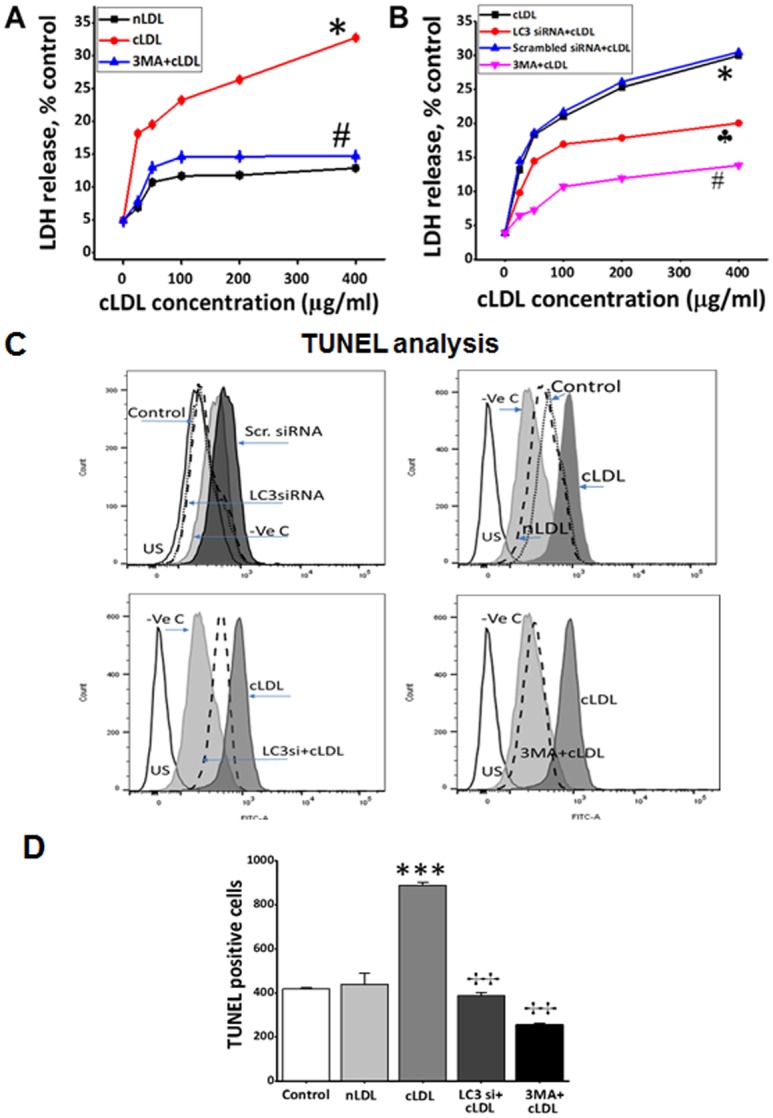
Effect of inhibition of cLDL-induced autophagy on cell death and DNA fragmentation in HCAE cells. **(A)** LDH release by cells after treatment with 5mM autophagy inhibitor 3MA and **(B)** LC3β siRNA transfection. (**A)** HCAE cells were treated with different doses of cLDL or nLDL with and without autophagy inhibitor 3MA for 8h as shown and media were collected from these cultures and analyzed for LDH release as described in Method section. **(B)** HCAE cells were transfected for 48 h LC3β siRNA or LC3β Scr. siRNA and treated with and without different doses of cLDL for 8h as shown. Cells were also treated with different doses of cLDL with and without autophagy inhibitor for 8h as shown. Following treatments, media were collected from these cultures and analyzed for LDH release as described in Method section. The data presented shows cLDL dose-dependence on LDH release in HCAE cells compared to untreated control cells. The expressed values are means ±SD. (n = 6). As shown, same doses of nLDL had no effect or very slight (~12%) on LDH release with the highest dose. Significantly (#*p*< 0.05) lower LDH release was recorded in the group when cells were pretreated with 5mM of 3MA before addition of cLDL, or transfected with LC3β siRNA (†*p* > 0.05), in comparison to cLDL- treated alone group. **(C)** Effect of autophagy inhibition on cLDL-induced DNA fragmentation in HCAE cells as measured by TUNEL assay. HCAE cells were transfected with LC3β siRNA/LC3β scr.siRNA as shown above followed by treatment with 100 μg/ml nLDL or 100 μg/ml cLDL for 8 h. After treatments with LC3β siRNA/LC3β scr.siRNA and cLDL (left panels), or CLDL/nLDL with and without autophagy inhibitor 3MA (right panels), apoptotic intensity of HCAE cells was determined by flow cytometry after TUNEL assay. Histograms shows the number (counts) of TUNEL positive cells in different groups as measured by flow cytometry. The overlapped peak demonstrates the effects as a whole. Data are shown as a logarithmic histogram and expressed as fluorescence intensity of number of counts of the TUNEL-positive cells obtained from the statistical analysis of the fluorescence height and mean value of the x-axis displayed by the software. For positive controls fixed and permeabilized cells treated with DNAse and negative controls without FITC labeling reagent were used. (D) Quantitative analysis of the counts of TUNEL-positive endothelial cells of each treatment is presented as the mean±SD of three independent experiments (***p <0.001 as compared to untreated controls, ††*p* <0.001 as compared to cLDL treated cells).

Treatment of HCAECs with cLDL significantly increased LDH release in a dose-dependent manner compared to that obtained with nLDL (Fig 5A). The autophagy inhibitor 3-MA markedly reduced a cLDL-induced increase in LDH release, indicating that inhibition of cLDL-induced autophagy prevents cell death in HCAECs. A genetic approach using LC3 siRNA was also used to examine the effect of autophagy inhibition on cLDL-induced cell death. Transfection of HCAECs with LC3 siRNA significantly prevented cLDL-induced LDH release compared to that of scrambled siRNA (Fig 5B). The effect of autophagy inhibition by using 3-MA and LC3 siRNA was also examined on DNA fragmentation using the TUNEL assay. Treatment of HCAECs with cLDL significantly increased the TUNEL-positive cells compared to nDL-treated or control cells. The autophagy inhibitor 3-MA or LC3 siRNA significantly prevented cLDL-induced TUNEL-positive cells (Fig 5C), indicating that autophagy inhibition prevented cLDL-induced DNA fragmentation. The quantification of the data for the TUNEL-positive cells is shown in Fig 5D. Taken together, these studies provide evidence that autophagy inhibition prevents cLDL-induced cell death and DNA fragmentation.

## Discussion

The present study provides evidence for the first time for the induction of autophagy and its role in endothelial cell (HCAEC) injury in response to cLDL. The autophagy response to cLDL was identified by specific induction of LC3-I, formation of lipid-conjugated LC3-II protein and formation of punctate staining of autophagosome-associated LC3-II. We demonstrated that autophagy is an immediate response to cLDL injury and autophagy inhibition by an autophagy inhibitor as well as by siRNA to LC3 provided protection from cLDL-induced cell death and DNA fragmentation. These studies provided evidence that autophagy is involved in cLDL-induced cell death and DNA fragmentation in endothelial cells.

Chronic kidney disease (CKD) is now recognized as an important independent risk factor for cardiovascular events, with progressive loss of kidney function being associated with higher morbidity and mortality [1]. However, the underlying mechanisms of this pathogenesis are not well understood. In CKD patients, a progressive decline in renal function results in an accumulation of uremic toxins in the blood. Although isocyanate or thiocyanate produced under uremic conditions lead to carbamylation of amino acids and proteins but carbamylation of LDL is most relevant to the development of atherogenesis. Some other modifications of LDL have been identified that are linked to atherosclerotic plaque formation and progression [30]. Recent studies suggest that cLDL is a dominant form of modified LDL that occurs either by urea-derived cyanate or thiocyanate-derived cyanate generated by the enzyme MPO (myeloperoxidase) [4]. Carbamylation rate of LDL is significantly increased in CKD patients [3,7,8,9].

Studies from our laboratory and that of others have implicated carbamylation as a potential major contributor to cardiovascular events in patients with chronic kidney disease (CKD). We have shown that cLDL is taken up by endothelial cells through specific receptors [13] and causes endothelial cell injury [7,31]. Other recent studies have shown that cLDL induced endothelial dysfunction including inhibition of eNOS phosphorylation and activation of endothelial LOX-1 receptor, which results in ROS production and eNOS uncoupling [32,11]. We have demonstrated that cLDL induces dose- and time-dependent DNA fragmentation [7], activates the MAPK pathway [33], causes vascular smooth muscle cell proliferation, and accelerates monocyte adhesion through activation of ICAM-1 and VCAM-1 adhesion molecules in endothelial cells [12]. These data suggest that cLDL has pro-atherogenic properties and may contribute to atherosclerosis in CKD. Moreover, recent studies provided evidence that serum levels of cLDL were elevated in CKD patients with cardiovascular disease [4,7,8,9]. Epidemiological studies demonstrated that carbamylated proteins are independent risk factors for morbidity and mortality in patients with CKD [9,11]. In diabetic patients on hemodialysis, serum carbamylated albumin was strongly associated with cardiac damage, risk of congestive heart failure, and sudden cardiac death (11). Carbamylation of LDL also occurs by myeloperoxidase-catalyzed oxidation of thiocyanate to cyanate independent of cyanate generated by dissociation of urea. Plasma levels of cLDL were elevated in patients with type 2 diabetes even with normal renal function [34] and the increased levels of cLDL were correlated with myeloperoxidase [34,35]. Although autophagy is known to play a critical role in diabetes [36,37], it will be of interest in future studies to determine the cause and effect relationship between cLDL and autophagy in diabetes.

At present the precise mechanisms underlying induction of autophagy by cLDL are not known. Previous studies have shown that cLDL causes oxidative stress and mitochondrial damage in human endothelial progenitor cells [38] and produces reactive oxygen species (ROS) in human umbilical vein endothelial cells [39]. cLDL-mediated oxidave stress may contribute to induction of autophagy since ROS and oxidative stress are known to induce autophagy [40]. In addition, in a recent study in cultured rat L6 muscle cells, cLDL decreased glucose uptake and glucose transporter 4 (GLUT4) to the membranes suggesting that cLDL may be involved in the development of type 2 diabetes and thus, it is possible that prevention of glucose uptake in endothelial cells may result in the induction of autophagy due to starvation. Therefore, future studies will examine the underlying mechanisms involved in the induction of cLDL-induced autophagy. Nevertheless, our studies provide evidence that autophagy is an important player in cLDL-mediated endothelial cell injury that may provide one of the underlying mechanisms for the pathogenesis of atherosclerosis.
